# The Cytoscan HD Array in the Diagnosis of Neurodevelopmental Disorders

**DOI:** 10.3390/ht7030028

**Published:** 2018-09-14

**Authors:** Francesca Scionti, Maria Teresa Di Martino, Licia Pensabene, Valentina Bruni, Daniela Concolino

**Affiliations:** 1 Department of Experimental and Clinical Medicine, Magna Graecia University, Salvatore Venuta Campus, 88100 Catanzaro, Italy; scionti@unicz.it (F.S.); teresadm@unicz.it (M.T.D.M.); 2 Department of Medical and Surgical Sciences, Pediatric Unit, Magna Graecia University, 88100 Catanzaro, Italy; pensabene@unicz.it (L.P.); valentina.bruni82@gmail.com (V.B.)

**Keywords:** copy number variations, SNP-array, neurodevelopmental disorders

## Abstract

Submicroscopic chromosomal copy number variations (CNVs), such as deletions and duplications, account for about 15–20% of patients affected with developmental delay, intellectual disability, multiple congenital anomalies, and autism spectrum disorder. Most of CNVs are de novo or inherited rearrangements with clinical relevance, but there are also rare inherited imbalances with unknown significance that make difficult the clinical management and genetic counselling. Chromosomal microarrays analysis (CMA) are recognized as the first-line test for CNV detection and are now routinely used in the clinical diagnostic laboratory. The recent use of CMA platforms that combine classic copy number analysis with single-nucleotide polymorphism (SNP) genotyping has increased the diagnostic yields. Here we discuss the application of the Cytoscan high-density (HD) SNP-array for the detection of CNVs. We provide an overview of molecular analyses involved in identifying pathogenic CNVs and highlight important guidelines to establish pathogenicity of CNV.

## 1. Introduction

A contribution to the human genome variability comes from copy number variations (CNVs), which involves unbalanced rearrangements, such as deletions and duplications, of intermediate size (>50 base pairs, bp). Higher resolution maps of CNVs estimated that 4.8–9.5% of the genome is involved in gain and losses which range in size from 300 to 3000 bp [1]. The spectrum phenotype of CNVs varies from normal to pathogenic conditions. It is now well-established that CNVs account for about 15–20% of patients affected with developmental delay (DD), intellectual disability (ID), multiple congenital anomalies (MCA), and autism spectrum disorder (ASD) [2]. These structural variants cannot be resolved by standard karyotype analysis because of its resolution limited to chromosomal aberrations greater than 3 Mb in size. In contrast to karyotype analysis, fluorescence in situ hybridization (FISH) and multiple ligation-dependent probe amplification (MLPA) are not genome-wide approaches as they have been developed to access copy number (CN) state of specific focused loci. The advent of microarrays has enhanced the capability to detect CNVs, discover new genomic syndromes and characterize breakpoints in phenotypically known disorders. The American College of Medical Genetics and Genomics (ACMG) [3], the International Collaboration for Clinical Genomics (ICCG) [4], and the American Academy of Neurology (AAN) [5] recommended chromosomal microarray analysis (CMA) as the first-line test in patients with unexplained DD/ID/MCA/ASD. There are currently different CMA platforms used in clinical practice which differ in technology, resolution and detection. Over the past 3–5 years, high-resolution CMA platforms that combine classic copy number analysis with single-nucleotide polymorphism (SNP) genotyping have identified many small CNVs that underlie neurodevelopmental disorders leading to increases in diagnostic yield for some of these patients [6]. Additionally, there may be differences in the clinical interpretation of CNV among laboratories. Most of CNVs are de novo or inherited rearrangements with clinical relevance, but there are also rare inherited imbalances with unknown significance that make difficult the clinical management and genetic counselling. In this review we discuss the application of Cytoscan high-density (HD) SNP-array in diagnostics providing an overview of its methodology and highlighting important guidelines to establish pathogenicity of CNV.

## 2. Chromosomal Microarray Platforms

DNA microarrays are collections of probes, complementary DNA (cDNA) fragments or oligonucleotides, spotted or directly synthesized in a high-density pattern onto a solid surface and developed to hybridize complementary target nucleic acids (genomic DNA or cDNA). This technology has been widely used to analyze the simultaneous expression profile of hundreds to thousands of genes, often in cancer samples [7]. More recent uses of DNA microarrays include the detection of methylation patterns [8], the molecular probe inversion genotyping of SNPs [9] and the detection of gene CN [10].

In particular, CMA platforms can be divided into two types: array-based comparative hybridization (array-CGH or a-CGH) and SNP-array; a-CGH is designed for the detection of CNVs using either bacterial artificial chromosome (BAC) or oligonucleotide probes. Bacterial artificial chromosome probes are approximately 150 kb in size and are less sensitive and provide less coverage respect to oligonucleotide probes (60–70 mer in size); a-CGH platforms use a two-color system in which the DNA test and DNA reference are labelled using different fluorophores (typically Cyanine 3 and Cyanine 5) and are hybridized to the same microarray. Copy number state of DNA test respect to DNA reference is calculated measuring the fluorescence ratio of each probe. Otherwise, SNP-array technology uses two types of probes (~25 bp): non-polymorphic probes for the detection of CNVs and polymorphic probes for allele genotype. While CN probes are designed to provide overall coverage of the genome, SNP probes are limited to specific genomic locations. Additionally, SNP genotyping allows an independent confirmation of CN finding. Short oligonucleotides (25-mer) provide a lower signal-to-noise ratio of hybridization than 60-mer probes and the analyses need to average several consecutive markers thus diminishing the overall resolution. In contrast, 25-mer probes are more specific allowing the discrimination of SNP under optimal conditions but with reduced sensitivity [11,12]. For SNP-arrays, a single sample is labelled and hybridized to the array and changes in CN are determined in silico, comparing the signal intensity of the sample with a set of analog experiments performed on hundreds of reference DNAs. a-CGH platforms may also be supplemented with SNP probes (Table 1). Although, SNP density on these arrays is typically lower than on the traditional SNP-arrays, a-CGH platforms with mid SNP density have shown similar performance [13]. However, in a clinical laboratory setting, some considerations need to be made when choosing a CMA platform. The ability of a platform to detect chromosomal imbalances is dependent on the resolution of a microarray that is directly correlated to the number, spacing and length of probes. An SNP-array has a higher resolution and better breakpoint determination with respect to other platforms due to greater probe number, smaller probe length and smaller probe spacings. This allows the identification of small and rare CNVs, but at the same time the assessment of the pathogenicity of these rearrangements requires more stringent parameters in order to avoid false positives [14]. Platforms which combine CN and SNP probes have the advantage of detecting long contiguous stretches of homozygosity (LCSH), defined as absence of heterozygosity (AOH), which might represent uniparental disomy (UPD), if confining to a single chromosome, or identity by descent consistent with parental consanguinity, if involving multiple chromosomes. In UPD both or parts of two homologous are transmitted by only one parent. In isodisomy two identical segments from one parental homologue are present, while in heterodisomy segments from both homologues are inherited from the same parent. The main mechanisms by which UPD occurs include meiotic non-disjunction with trisomy or monosomy rescue, gamete complementation and somatic recombination. Uniparental disomy may have clinical relevance if the LCSH segment contains imprinted genes, resulting in imprinting disorders such as Prader–Willi syndrome and Angelman syndrome [15]. Moreover, in isodisomy the two identical segments may have an autosomal recessive mutation, inherited from a carrier parent, resulting in a recessive genetic disease in the proband. It is estimated that a normal individual might have between 20 and 150 Mb of homozygosity involving between 1 and 5 Mb of DNA in any stretch [16]. Long contiguous stretches of homozygosity suggest UPD when greater than 8 Mb if telomeric and greater than 15 Mb if interstitial [16]. It is important to consider that using SNP information, CMA platforms can identify only isodisomy or combinations of isodisomy and heterodisomy while fails to detect complete heterodisomy [17]. Isodisomy is easily detectable due to the complete absence of heterozygosity along the entire length of a chromosome. Heterodisomy does not contain LSCH and cannot be distinguished from regions with normal biparental inheritance without trio analysis. However, in many cases of UPD, there is a detectable mixture of regions of iso- and heterodisomy generated by meiotic recombination. 

In consanguinity, chromosomal segments result identical by descent (IBD) and the number and size of AOH segments correlate with the degree of relatedness. The percentage of IBD is calculated by dividing the total length of LCSH in autosomes (X and Y chromosomes are excluded because males are hemizygous), with a size greater than 3 Mb, and the total length of autosomes (2,867,733 kb for hg18) [18]. Typically, in clinical laboratories, LSCH with a size below 3 Mb are not considered significant [19]. A percentage of IBD greater than 10% correlates with first- or second-degree parental relationship inducing suspicion of abuse in proband’s mother, especially when she is affected by ID or is a minor. Also, the analysis of homozygous regions could be extremely useful for the identification of candidate genes when there is the suspect of autosomal recessive disorders. Therefore, AOH information, in addition to that provided from CN in the same platform, maximizes the diagnostic yield from an array testing [20]. Mosaicism can be detected using a-CGH platforms with a minimal detection of 10–20% [21]. However, mosaicism at levels as low as 5% has been reported using platforms which combine CN and SNP probes [22]. One of the major limitations of array-based technologies is the inability to detect balanced translocations such as Robertsonian or other reciprocal translocations, insertions or balanced inversions. However, about 30–40% of cytogenetic events which appear balanced on the microscopic level, have a submicroscopic imbalance when tested with high-resolution array technology, above all SNP-array [23,24].

## 3. Cytoscan HD Platform: An Overview

The CytoScan HD array was launched by Affymetrix (Affymetrix, Santa Clara, CA, USA), now part of Thermo Fisher Scientific (Thermo Fisher Scientific, Inc.; Waltham, MA, USA) for CN analysis. This technology includes 1.9 million CN markers and 750,000 genotype-able SNPs. The average marker spacing in intragenic regions is 880 bp, covering 100% of Online Mendelian Inheritance in Man (OMIM) genes, 98% of RefSeq genes and 100% of Sanger cancer genes, while in intergenic regions is about 1737 bp.

**Figure 1:**
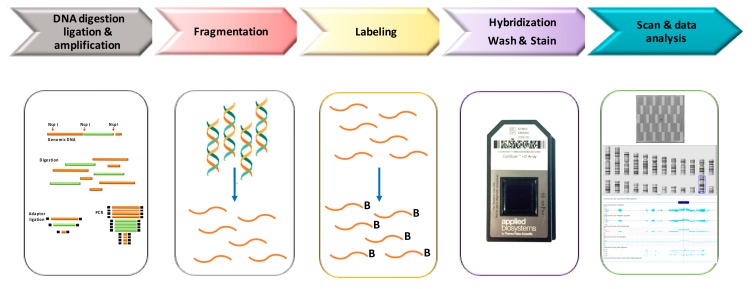
Workflow of Cytoscan High-density (HD) analysis.

In Figure 1, we have depicted a schematic laboratory workflow of Cytoscan HD assay. Genomic DNA (250 ng) is digested by NspI and amplified using a ligation-mediated PCR with adapters covalently linked to the restriction fragments. In the next step PCR products are purified using magnetic beads, fragmented using DNase I, labeling with biotin and hybridized overnight (16–18 h) to a 49-format array. After incubation samples are washed and stained with streptavidin using a GeneChip Fluidics Station 450. Finally, arrays are scanned by GeneChip Scanner 3000, using the GeneChip Command Console Software (Thermo Fisher Scientific), to generate the CEL files that includes the intensity probe signals.

**Figure 2:**
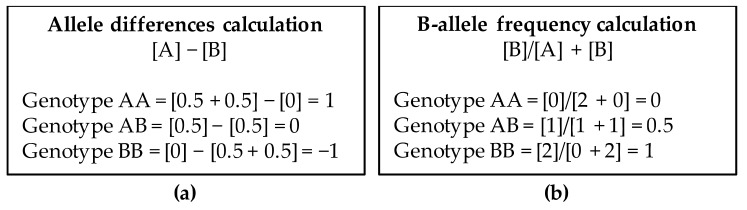
Genotype values in Allele difference plot (**a**) and B-allele frequency plot (**b**).

CEL files are analyzed using the Chromosome Analysis Suite (ChAS) software (Thermo Fisher Scientific, Inc) and converted to CYCHP files containing information on copy number, loss of heterozygosity (LOH), mosaicism, and genotype calls. Copy number state and SNP genotypes are called using the Hidden Markov Model (HMM) algorithm and the Bayesian Robust Linear Model with the Mahalanobis distance classifier (BRLMM) algorithm, respectively. The intensity ratio of each SNP and CN probe in the DNA test provide a relative copy number (log2 ratio: log2_sample_ − log2_reference_) which is normalized respect to a reference. The Reference Model File contains 380 samples, 284 from HapMap and 96 from BioServe Biotechnologies (BioServe Biotechnologies, Ldt; Beltsville, MD, USA). Determination of log2 ratio will indicate if there is a gain or loss of genetic material. The alleles corresponding to the nucleotide base change are given the designation of allele A and allele B; SNP genotypes are visualized using both the allele difference plot and the B-allele frequency plot (BAF). The allele difference is calculated as the difference between the number of the two alleles using the formula [A] − [B]. Each allele has a value of 0.5. B-allele frequency is the number of the B alleles divided by the total number of alleles of a SNP (AA, BB or AB) and is calculated with the formula: [B]/[A] + [B]. Genotype value calculations for both type of plots are shown in Figure 2.

**Figure 3:**
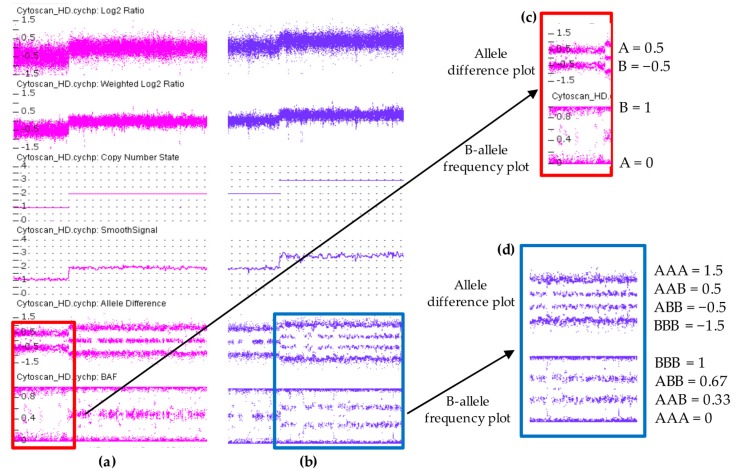
Copy number variations analysis using ChAS 3.3 software displaying a hemizygous deletion in (**a**) and a hemizygous duplication in (**b**). Each dot represents a single SNP in the array. Deletion is associated with loss of signal intensity consistent with a decrease in Log2 ratio, Weighted Log2 ratio, CN state and smooth signal. In contrast, duplication region shows increased values. Allele difference plot and B-allele frequency (BAF) plot of copy number variations (CNVs) regions are designated within the highlighted boxes. In the deletion both plots show two tracts (A and B) instead of three indicating a presence of a single allele (**c**). These two tracts are at 0.5 and −0.5 in allele difference plot while in BAF plot are at 0 and 1. In duplication, the allele difference and the BAF plots show four tracts (AAA, AAB, BBA, and BBB) instead of three (**d**). These four tracts are at 1.5, 0.5, −0.5, −1.5 in allele difference plot, while in BAF plot are at 0, 0.33, 0.67, 1.

In a sample with a diploid genome, the CN state is equal to two, the log2 ratio is equal to zero [Log2(2/2) = 0] and allele difference plot and BAF plot showing the AA, AB and BB allele tracts for the three normal genotypes according to the genotype values reported in Figure 2. In contrast, when there is a deletion region, all the probes in the log2 ratio are centered on the −0.45 line, while at the same time the allele difference plot and BAF plot show two tracts (A, B) instead of three, indicating a presence of a single allele (Figure 3c). In a sample with a duplication, all of the probes in the log2 ratio are centered on 0.3 line and the allele difference and the BAF plots show four tracts (AAA, AAB, BBA, and BBB) instead of three (Figure 3d). The log2 ratio values of −0.45 and 0.3 differ from the theoretical values of −0.1 [=log2(1/2)] and 0.58 [=log2(3/2)] calculated for deletion and duplication, respectively. These values were established by Affymetrix analyzing a dataset of 1400 samples with well-characterized CN changes across 75% of the genome.

Two other probe array data displayed by ChAS are the Weighed Log2 ratio and the Smoothed Signal. The Weighed Log2 is a measure of the log2 ratios processed through a Bayes wavelet shrinkage estimator. These processed values are input to the CNState algorithm HMM. The Smoothed Signal is used to estimate CN by Gaussian smoothing and allele peaks are inferred using a nonparametric estimation of filtered and smoothed values of individual probe. The LOH, which results in a tract with runs of homozygosity (ROH), is detected examining allele patterns across all chromosomes. Loss of heterozygosity is visualized when there is a DNA segment of homozygous genotypes of about 1 Mb stretch or greater. In UPD/LOH/mosaicism the AA, AB, and BB signals shift away from the integer state corresponding to a non-mosaic CN state. 

## 4. Clinical Applications of Cytoscan HD Array in Neurodevelopmental Disorders

Although Cytoscan HD array has been extensively used in prenatal diagnosis of chromosomal abnormalities and neoplastic samples [25,26], we focused on neurodevelopmental disorders, looking in particular to large-scale studies that have employed this platform both as unique tool and in combination with other platforms (Table 2). Pereira et al. [27] used Cytoscan HD to perform a study on 15 ID patients with normal karyotype analysis and negative X-fragile test. The rate of pathogenic CNV was 26.7%. Recently, a similar percentage (25%) was reported by Wang et al. [28] in a group of 489 ID patients analyzed with the same platform. These diagnostic yields are higher if compared with studies that have employed previous platforms [29,30], demonstrating the increased resolution provided by Cytoscan HD. Zarrei et al. [31] using Cytoscan HD found nine de novo CNV in 7/97 (7.2%) individuals affected by hemiplegic cerebral palsy involving important developmental genes (*GRIK2*, *LAMA1*, *DMD*, *PTPRM*, and *DIP2C*). Al-Qattan et al. [32] analyzed a cohort of 183 DD/ID patients in consanguineous population of Saudi Arabia using three SNP-array platforms (Cytoscan HD, Affymetrix SNP6.0 and Cyto-V2). The authors identified 40 pathogenic CNVs in 38 patients with an overall relatively high yield (21%). Asadollhai et al. [33] investigated the clinical significance of small CNV (<500 bp) in 714 patients with neurodevelopmental disorders using three different platforms (CytoScan HD, 212 patients; Affymetrix Genome-Wide Human SNP Array 6.0, 79 patients; Affymetrix Cytogenetics 2.7, 423 patients). The diagnostic yield was similar between Cytoscan HD (3.3%) and Cytogenetics 2.7 (3.5%), while it was higher for SNP 6.0 (5.1%). In overall, they found pathogenic or likely pathogenic CNVs in 2.2% of cases. This percentage is slightly lower than the ~3% observed by Hollenbeck et al. [34] in a cohort of 4417 patients referred to CMA. Additionally, Fan et al. [35] reported three partial deletions of *AUTS2* gene in three patients with unexplained DD/ID. This gene is known to be associated with ID, DD, ASD, neurological abnormalities, short stature, microcephaly and facial dysmorphism. They found two de novo heterozygous deletions involving exon 6 (98.4 kb and 262 kb) and one spanning 12–19 exons (2147 kb) at the C-terminal of *AUTS2*, and few other genes near to the William–Beuren syndrome critical region. They demonstrated the high-resolution provided by Cytoscan HD and the ability to detect small intragenic deletions.

## 5. Clinical Interpretation of Copy Number Variations

In 2011 the ACMG published a document including standard and guidelines for interpretation of postnatal constitutional CNVs [36]. Table 3 reports the current clinical classification of CNVs and their description according to ACMG.

**Figure 4:**
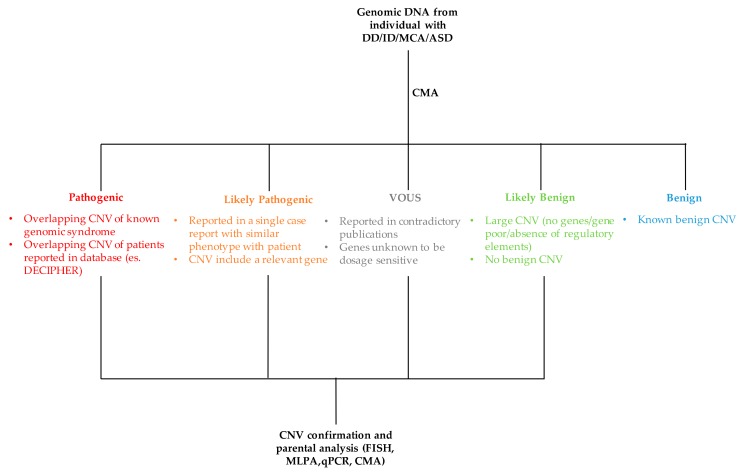
Flowchart of CNV interpretation process. MCA: multiple congenital abnormalities, ASD: autism spectrum disorder, MLPA: multiple ligation-dependent probe amplification, FISH: fluorescence in situ hybridization, qPCR: quantitative PCR.

The process of classifying a CNV as either pathogenic or benign is not simple and straightforward as it requires the evaluation and integration of several data (Figure 4). The major criteria used for the interpretation of CNV are discussed below.

- *Copy number variations size*. Although there is a positive correlation between the increase of CNV size and its clinical relevance, this is not to be taken as a general rule. Large CNVs have been described as polymorphisms as otherwise small CNVs involving a single gene can be pathogenic.
- *Gene content*. The gene content of a CNV should be carefully evaluated for clinical association with the phenotype of proband. One should be verified if a gene or a group of genes, included in a duplication or deletion, are dosage-sensitive and associated with diseases. In this process, some considerations are important. First, if a gene is reported to be associated with a clinical phenotype when deleted or mutated, the duplication of the same gene may have no clinical relevance. Also, intragenic duplications may be pathogenic altering coding sequence, in contrast intronic deletions may have no clinical effect. If no mutation is reported in clinical literature for a gene, then it is recommended to avoid any conclusion of pathogenicity only on the basis of in silico analysis or in vitro and/or animal studies. A deletion of a gene associated with an autosomal recessive disorder may suggest the presence of a mutation on the second allele. Moreover, a CNV without genes in its interval generally is not reported in clinical laboratories. Another consideration is on CNV confirmation. Small deletions and duplications can be confirmed using quantitative-PCR (qPCR) and MLPA, while large CN (deletions >150 kb and duplications >400 kb) can be validated by other technique such as FISH and microarray. Despite the majority of duplications are in tandem, in a subset of cases the duplicated material resides on a different chromosome or in an atypical location on the chromosome of origin due to an unbalanced translocation or an inversion. In this context, FISH analysis is useful for a better characterization of the underline mechanism and for appropriate recurrence risk calculation.
- *Databases*. The are many public catalogs available for CNV interpretation. Among these the most used are the Database of Genomic Variants (DGV; http://dgv.tcag.ca/dvg/app/home), the Database of Chromosomal Imbalance and Phenotype in Human using Ensembl Resource (DECIPHER; https://decipher.sanger.ac.uk) and the Clinical Genome Resource (ClinGen; https://www.clinicalgenome.org). The DGV include human genomic structural variations found in healthy individuals and collected from worldwide studies. Although not present in ACMG recommendations, some authors suggest considering a CNV benign if present in at least three control individuals with the same orientation (deletion/duplication) [37]. The DECIPHER contains data from patients including both clinical phenotypes and genomic rearrangements. The ClinGen is a National Institutes of Health (NHI)-funded resource of clinically annotated genes and variants for use in precision medicine and research. ClinGen has a curated genome-wide dosage sensitivity map which can be used for the clinical interpretation of CNV. This resource provides evidence-based correlations between haploinsufficiency (loss) or triplosensitivity (gain) of a gene or genomic regions and clinical phenotypes. In addition, ClinGen provides CNV data from contributing laboratories and their classification, displayed in the NCBI ClinVar database. Finally, in-house or national reference database could be useful to construct a CNV map characterizing regional populations.
- *Parental analysis*. The inheritance of a CNV by an affected parent may support its pathogenicity. However, this event may be coincidental. When available, the evaluation of additional familial members may be useful to verify if the CNV continues to segregate with the phenotype. The inheritance of a CNV by an unaffected parent may not exclude its pathogenicity due to incomplete penetrance, variable expression, parent of origin imprinting effects or mosaic CNV in parent. Also, as reported above, the occurrence of an autosomal recessive disorder should be taken into consideration.

## 6. Conclusions

High-resolution CMA such as CytoScan HD have improved the ability to identify CNVs undetectable by other technologies such as karyotyping, FISH, and targeted or lower-resolution array platforms due to lower resolution and/or coverage. Additionally, the diagnostic yield of this platform is enhanced by the detection of LCSH, which are accessed genotyping of thousands of SNPs, and are suggestive of either UPD or increased risk of recessive conditions. The performance of this technology to identify gains and losses in patients with DD/ID/MCA/ASD has been well documented, but its applicability has been also reported in neoplastic samples and prenatal specimens. However, the increased yield in detecting potential clinically relevant small and rare CNVs (<500 kb), affecting single or few genes, raises the problem regarding how to interpret these variants and the need to their validation in order to avoid false positive results. The pathogenicity of a CNV remains challenging and consequently requires the integration of several data for an accurate interpretation which include inheritance, biological function of gene content, and comparison with public databases. In particular, the availability of larger submitted and shared genomic and clinical data could improve this process.

## Figures and Tables

**Table 1:** Chromosomal microarray analysis (CMA) platform comparison.

SNP-array	a-CGH	a-CGH CN + SNP
Oligonucleotide probe length: ~25 bp	Oligonucleotide probe length: 60–70 bp	Oligonucleotide probe length: 60–70 bp
Copy number probe + SNP probe (high density)	Copy number probe only	Copy number probe + SNP probe (low or mid density)
Hybridization of DNA test only	Hybridization of DNA test and DNA reference	Hybridization of DNA test and DNA reference
Detection of UPD and consanguinity	No detection of UPD and consanguinity	Detection of UPD and consanguinity

SNP: single nucleotide polymorphism, a-CGH: array-based comparative genomic hybridization, CN: copy number, UPD: uniparental disomy.

**Table 2:** Studies reporting the use of Cytoscan HD array alone or in combination with other SNP-array platforms.

Reference	Patients (*n*)	Disorder	CMA Platform	CNV Size (kb)	Origin		CNV Interpretation		
					De Novo *n* (%)	Inherited *n* (%)	Pathogenic *n* (%)	Lakely Pathogenic *n* (%)	VOUS *n* (%)
Pereira et al. [27]	15	ID	Cytoscan HD	≥100	9 (50)	9 (50)	4 (22)	4 (22)	10 (56)
Wang et al. [28]	489	ID	Cytoscan HD	≥100	141 (70%)	60 (30%)	122 (61%)	4 (2)	75 (37)
Zarrei et al. [31]	97	CP	Cytoscan HD	≥10	9 (30)	21 (70)	4 (13.3)	1 (3.3)	25 (83.4)
Al-Qattan et al. [32]	183	DD/ID	Cytoscan HD	≥200	40 (90) *	4 (10) *	40 (81.6)	5 (10.2)	4 (8.2)
			Affymetrix SNP Array 6.0						
			Cyto-V2						
Asadollhai et al. [33]	714	NDD	Cytoscan HD	<500	12 (46.1)	14 (53.4)	12 (46.1%)	4 (15.4)	10 (38.5)
			Affymetrix SNP Array 6.0						
			Affymetrix Cytogenetics 2.7						

* Five cases were of unknown inheritance. ID: intellectual disability, DD: developmental delay, CP: cerebral palsy, NDD: neurodevelopmental disorder, VOUS: variant of uncertain significance.

**Table 3:** CNV classification according to American College of Medical Genetics and Genomics (ACMG).

CNV Classification	Description
**Pathogenic**	The CNV is documented as clinically significant in multiple peer-reviewed publications, even if penetrance and expressivity of the CNV are known to be variable
**Benign**	The CNV has been reported in multiple peer-reviewed publications or curated databases as a benign variant, particularly if the nature of the copy number variation has been well characterized and/or the CNV represents a common polymorphism
**Uncertain clinical significance CNV (Likely pathogenic)**	The CNV is described in a single case report but with well-defined breakpoints and phenotype, both specific and relevant to the patient findings.
	A gene within the CNV interval has a very compelling gene function that is relevant and specific to the reason for patient referral
**Uncertain clinical significance CNV (Likely benign)**	The CNV has no genes in interval but exceeds a size criterion that may be established by the laboratory.
	The CNV is described in a small number of cases in databases of variation in the general population but does not represent a common polymorphism
**Uncertain clinical significance CNV (No subclassification)**	The CNV contains genes, but it is not known whether the genes in the interval are dosage sensitive.
	The CNV is described in multiple contradictory publications and/or databases, and firm conclusions regarding clinical significance are not yet established
